# F-*O*-G Ring Formation in Glycopeptide Antibiotic Biosynthesis is Catalysed by OxyE

**DOI:** 10.1038/srep35584

**Published:** 2016-10-18

**Authors:** Madeleine Peschke, Clara Brieke, Max J. Cryle

**Affiliations:** 1 Department of Biomolecular Mechanisms, Max Planck Institute for Medical Research, Jahnstrasse 29, 69120 Heidelberg, Germany; 2 EMBL Australia, Monash University, Clayton, Victoria 3800, Australia; 3 The Monash Biomedicine Discovery Institute, Department of Biochemistry and Molecular Biology and ARC Centre of Excellence in Advanced Molecular Imaging, Monash University, Clayton, Victoria 3800, Australia

## Abstract

The glycopeptide antibiotics are peptide-based natural products with impressive antibiotic function that derives from their unique three-dimensional structure. Biosynthesis of the glycopeptide antibiotics centres of the combination of peptide synthesis, mediated by a non-ribosomal peptide synthetase, and the crosslinking of aromatic side chains of the peptide, mediated by the action of a cascade of Cytochrome P450s. Here, we report the first example of *in vitro* activity of OxyE, which catalyses the F-*O*-G ring formation reaction in teicoplanin biosynthesis. OxyE was found to only act after an initial C-*O*-D crosslink is installed by OxyB and to require an interaction with the unique NRPS domain from glycopeptide antibiotic – the X-domain – in order to display catalytic activity. We could demonstrate that OxyE displays limited stereoselectivity for the peptide, which mirrors the results from OxyB-catalysed turnover and is in sharp contrast to OxyA. Furthermore, we show that activity of a three-enzyme cascade (OxyB/OxyA/OxyE) in generating tricyclic glycopeptide antibiotic peptides depends upon the order of addition of the OxyA and OxyE enzymes to the reaction. This work demonstrates that complex enzymatic cascades from glycopeptide antibiotic biosynthesis can be reconstituted *in vitro* and provides new insights into the biosynthesis of these important antibiotics.

The glycopeptide antibiotics (GPAs) – which include the compounds vancomycin and teicoplanin – are highly complex heptapeptide natural products with impressive activity against Gram-positive bacteria^1^. Produced by a non-ribosomal peptide synthetase (NRPS) (Fig. 1)^2^,^3^, the GPA precursor peptide is rich in non-proteinogenic amino acids, and in particular phenylglycines, which stems from the ability of NRPSs to utilise a wide range of substrate amino acids for their construction^4^,^5^. The source of GPA activity – binding to the dipeptide terminus of lipid II, thus preventing the correct crosslinking of the peptidoglycan cell wall – is enabled by their highly specific, rigid three-dimensional shape^1^,^6^. This structure is obtained through the crosslinking of the side chains of aromatic residues contained within the peptide, which is performed by the actions of several Cytochrome P450 monooxygenases (Fig. 1)^7^,^8^. As powerful oxidative catalysts, these enzymes play many roles in the biosynthesis of natural products, which is due to their ability to perform challenging oxidation reactions such as hydroxylation, epoxidation and aromatic crosslinking with high degrees of selectivity^9^. Given that the GPA peptide crosslinking cascade is the source of both their activity and their general intractability to synthesis at scale by chemical means^10^,^11^,^12^, there is great interest in understanding this process.

GPAs can generally be divided into different classes based upon their amino acid composition and the number of crosslinks found within their final structure: type I GPAs such as vancomycin exhibit three crosslinks in their structure (known as the AB, C-*O*-D and D-*O*-E rings) and possess aliphatic/polar residues at positions one and three of the peptide, whilst type IV GPAs such as teicoplanin exhibit an additional ring in their structure (the F-*O*-G ring). The formation of the F-*O*-G ring is enabled by the presence of aromatic residues at positions one and three of the peptide and an additional P450 enzyme (Fig. 1)^1^. Many studies using *in vivo* and *in vitro* techniques have served to contribute to our greater understanding of this cascade, along with their potential to act as biocatalysts. In particular, *in vivo* studies have shown that each of the P450 enzymes – known as the Oxy enzymes – catalyse the installation of a specific ring within the GPA peptide, and that there is a general order to their installation, which commences with the installation of the C-*O*-D ring performed by OxyB^13^,^14^,^15^,^16^,^17^,^18^. Within the simpler type I GPAs this is followed by the installation of the D-*O*-E ring, catalysed by OxyA, and finally the installation of the AB ring, which is performed by OxyC (Fig. 2b)^14^,^15^,^16^,^17^,^18^. In case of type IV GPAs, the additional ring that is installed by the enzyme OxyE has been postulated to occur before to OxyA, which was inferred from a detailed analysis based upon gene disruption experiments (Fig. 2a)^13^. *In vivo* experiments also provided early evidence for the role of the peptide synthesis machinery – the NRPS – in the crosslinking activity of the Oxy enzymes^15^. In addition, mutasynthesis studies provided clear evidence for the ability of the Oxy enzymes to tolerate modified peptides (Fig. 2c)^19^,^20^. In particular, one of the most interesting examples of the flexibility of the Oxy enzymes was the installation of an alternate, larger AB ring into the final GPA aglycone, although the resultant product was no longer active as an antibiotic (Fig. 2c)^15^.

*In vitro* studies have also added a great deal to our understanding of this process, first of all demonstrating that carrier protein (CP)-bound substrates were required as substrates for the Oxy enzymes^21^,^22^,^23^,^24^,^25^,^26^, and most recently showing that a further NRPS domain – known as the X-domain – was in fact the recruitment element needed for efficient Oxy activity against CP-bound peptides *in vitro*^27^,^28^,^29^,^30^; this also agrees with data from *in vivo* experiments^31^. The ability to identify the X-domain as the missing link in Oxy activity has enabled rapid progress in both understanding the mechanism of the GPA oxidative cascade as well as the reconstitution of the Oxy step in GPA cyclisation^27^,^28^,^29^,^30^. In this work, we are able to report the first *in vitro* activity of OxyE, the F-*O*-G ring installing enzyme from the biosynthesis of type IV GPAs, along with studying its interaction with the NRPS. Furthermore, we have been able to combine this enzyme with OxyB and OxyA to show tricyclisation of a GPA precursor peptide and demonstrate that OxyE activity occurs before that of OxyA.

## Results

### Role of the NRPS machinery in OxyE recruitment to the peptide substrate

We first set out to clarify the role of the NRPS machinery in the reaction catalysed by OxyE. Previous results had indicated a lack of binding of linear *peptidyl*-PCP substrates to OxyE^32^, which tallies with *in vivo* results that OxyB activity is a prerequisite for the activity of subsequent Oxy enzymes in the cyclisation cascade^13^. Initial characterisation of the X-domain as the recruitment partner for OxyA, OxyB and OxyC had shown no interaction with OxyE^28^. This was puzzling since the X-domain interaction interface determined on the Oxy protein surface is conserved between the crosslinking Oxys. However, in recent *in vitro* investigations of the A47934 system^33^ we have noted that Oxy/NRPS interactions can be susceptible to the salt concentration used in such gel filtration-based studies^30^. This is in good agreement with the interaction interface between the Oxy proteins and the X-domain being composed largely of ionic or polar interactions as shown in the complex structure of OxyB and the X-domain^28^. Hence, we then tested lower salt concentrations for our interaction studies with OxyE and the X-domain, both from the teicoplanin^34^,^35^ system. Under these conditions, we now could see evidence for complex formation between OxyE and the X-domain, which indicates that the same general recruitment mechanism also occurs with OxyE (Fig. 3).

### Reconstitution of *in vitro* OxyE cyclisation activity

With interaction studies indicating that X-domain mediated recruitment was likely to also be needed for the activity of OxyE, we commenced investigations of *in vitro* turnovers of OxyE (SI Table 1). Initially, we tested the hypothesis based on *in vivo* data that OxyB activity – and hence the presence of the C-*O*-D ring – was necessary for OxyE activity^13^. We therefore performed turnover of a model teicoplanin heptapeptide (T7P(d/l-Hpg_7_) loaded onto both the isolated PCP domain 7 from the final NRPS module of the teicoplanin biosynthesis and the di-domain construct comprising the PCP- and X-domain from the same module: the peptide loading was accomplished using the promiscuous phosphopantetheinyl transferase Sfp^36^ and the *peptidyl*-CoA conjugate that was prepared *via* our reported Fmoc-solid phase synthetic route^27^,^28^,^29^,^37^. The results of OxyE turnover using appropriate redox partners (see below) of both substrate constructs was – as anticipated – extremely limited with only a few percent conversion of the linear peptide (Fig. 4a).

**Figure 2:**
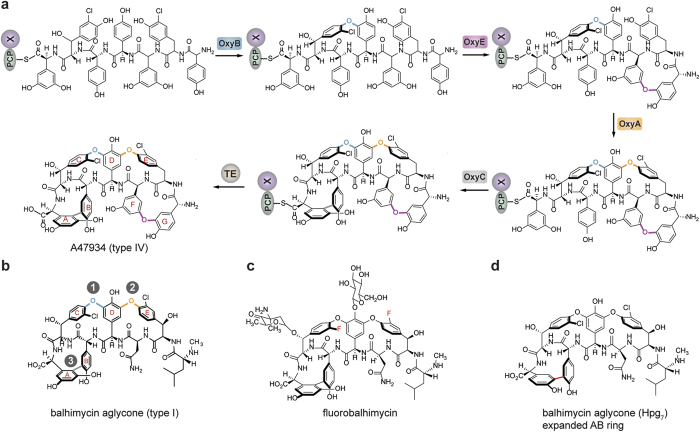
The cyclisation order of the teicoplanin-like type IV GPA A47934 as derived from *in vivo* gene disruption experiments showing the rings installed by each Oxy enzyme (**a**) along with the results for the type I GPA balhimycin (**b**); examples of altered GPAs isolated from mutasynthesis experiments, including fluorobalhimycin (**c**) and balhimycin with an expanded AB ring (**d**).

**Figure 3:**
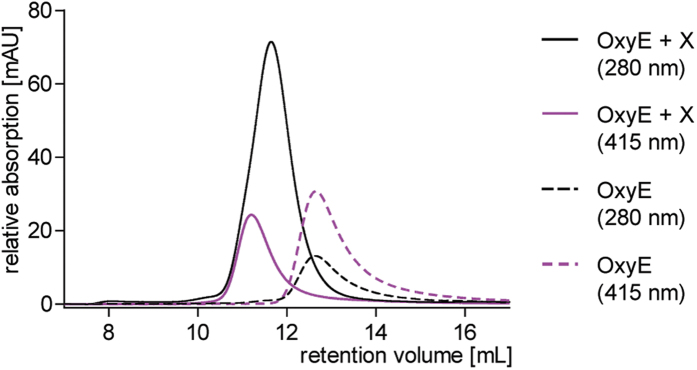
Gel filtration based co-elution experiments demonstrate that OxyE binds to the X-domain: this is demonstrated by the shift in OxyE heme absorbance (415 nm) to smaller retention volumes upon incubation with the X-domain, which indicates increased molecular weight and hence complex formation between the X-domain and OxyE. AU, arbitrary units.

**Figure 4:**
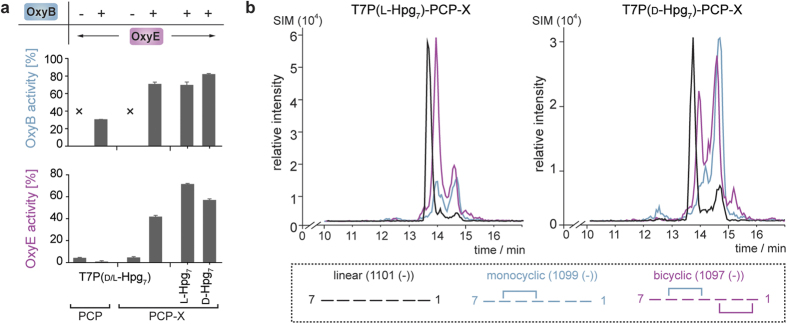
Activity of OxyE depends upon both the presence of the X-domain and the C-*O*-D ring within the PCP-bound peptide substrate, but shows limited selectivity for the correct peptide diastereomer at residue 7 of the peptide (**a**); traces from coupled OxyB/OxyE turnover experiments against PCP-X bound peptide substrates (**b**).

**Figure 5:**
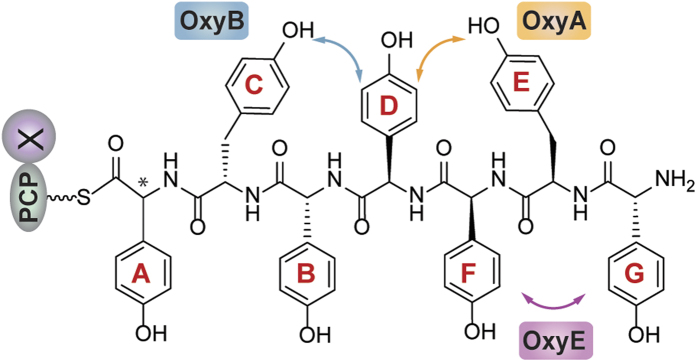
Peptide structure used in this study together with the sites of Oxy activity against this peptide.

We then performed the same experiments with the inclusion of OxyB, which has activity for both PCP and PCP-X constructs, albeit with a strong preference for oxidation of the peptide substrate coupled to the di-domain construct^21^,^28^. Given the requirement to provide the Oxy enzymes with a functional electron transfer chain, we tested a variety of redox partners to identify those best able to support Oxy activity: the systems tested included ferredoxin/ferredoxin reductase pairs PdR/Pdx^38^, ArR/Arx^39^, HaPuR/HaPux^40^, PuR/Pux^41^, PuR/PuxB^41^, the commonly utilised system FdR/Fdx from spinach and the pairing of *E. coli* FdR with flavodoxin Cdx^42^,^43^. We identified the most effective electron transport pair as palustrisredoxin reductase (PuR)/palustrisredoxin B (PuxB variant A105V) from *Rhodopseudomonas palustris*^41^; yields of cyclised products from related systems (ArR/Arx, HaPuR/HaPux, PdR/Pdx, PuR/Pux) were slightly reduced to those of PuR/PuxB, whilst SpR/Spx and FdR/Cdx supported turnovers were significantly lower. The results obtained from coupled OxyB/OxyE turnover experiments indicate that, as expected, OxyB activity proceeded to over 60% of the peptide presented on the di-domain PCP-X construct, and one third of these levels when the peptide was presented by the isolated PCP-domain (Fig. 4a). Crucially, we could observe significant levels of OxyE activity (40% conversion) of the monocyclic peptide produced by OxyB only when the substrate was presented by the PCP-X di-domain construct; no OxyE cyclisation activity against the PCP-loaded monocyclic peptide was detected, which clearly indicates the importance of the X-domain in OxyE recruitment and hence cyclisation activity of this enzyme (Fig. 4a). In discussing the activity of OxyE in cyclising the model peptide substrate T7P(d/l-Hpg_7_) it should be noted that this peptide contains an alternate residue at position three relative to the natural peptide substrate, where 3,5-dihydroxyphenylglycine (Dpg) is replaced by 4-hydroxyphenylglycine (Hpg) due to synthetic challenges in preparing the Dpg-containing peptide (Fig. 5). The ability of OxyE to still cyclise this peptide substrate is reminiscent therefore of the reports of OxyC activity against a similarly altered peptide *in vivo* (Fig. 2c), and again serves to indicate the potential flexibility of these P450 enzymes in cyclising alternate peptide substrates^15^; a tetracyclic GPA gene cluster has even been reported in which position three of the peptide is believed to be an Hpg residue^44^. As OxyA has been shown to be highly selective for the correct stereochemistry of residue seven of the substrate monocyclic peptide^27^, we also tested the incubation of OxyB and OxyE with individual peptide diastereomers T7P(l-Hpg_7_) and T7P(d-Hpg_7_). The results of turnover show that the natural l-diastereomer is preferred by OxyE, although the level of discrimination over the non-natural d-diastereomer is far less pronounced than for OxyA (by a factor of ~2)^27^ (Fig. 4).

### OxyE competes for NRPS substrates together with other Oxy enzymes

**Figure 6:**
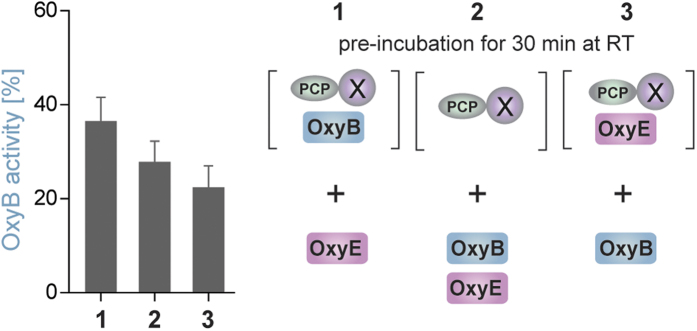
Results of single turnover experiments using OxyB in competition with OxyE that indicate OxyE competes with OxyB for binding to the linear *peptidyl*-PCP-X construct in the same manner as has been observed for OxyA and OxyC.

Given that OxyE activity was dependent upon the presence of the X-domain within the peptide-presenting substrate, we wanted to determine whether the X-domain interface that interacts with OxyE was the same as we had determined for the other Oxy enzymes. To achieve this, we performed competition studies together with OxyB under single turnover conditions and limiting concentrations of *peptidyl*-PCP-X substrate. Using alternate orders of pre-incubation of the two Oxy enzymes (OxyB before OxyE (1), simultaneous addition of OxyB and OxyE (2), OxyE before OxyB (3)), we could observe a decrease in OxyB cyclisation activity with OxyE addition as shown in Fig. 6 (also see SI Table 2). These results also clearly demonstrate competition for the same X-domain binding site between OxyB and OxyE, with the OxyB pre-incubation sample providing the highest levels of cyclisation and the OxyE pre-incubation sample providing the lowest levels of cyclisation (Fig. 6). These results are in line with data obtained for OxyA and OxyC, although OxyE appears to be the least able to compete with OxyB as indicated by higher levels of activity in all samples when compared to OxyA/C^27^. This result not only fits well with the conserved nature of the residues in the Oxy proteins and those shown to be important for the OxyB/X-domain complex^28^, but also matches the results of interaction studies that indicate that OxyE has a weaker interaction with the X-domain when compared to the other Oxy enzymes.

### Reconstitution of GPA peptide tricyclisation using an *in vitro* cascade of Oxy enzymes

**Figure 7:**
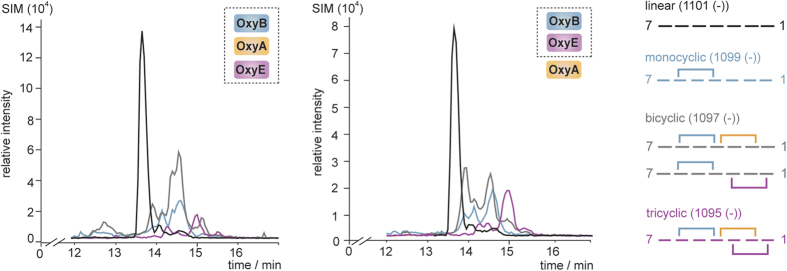
LCMS traces from Oxy-catalysed turnover of T7P(l-Hpg_7_) PCP-X substrates using an OxyB/OxyA/OxyE cascade (left hand side) or OxyB/OxyA/OxyE cascade with initial OxyB/OxyE incubation (right hand side); trace colours: black – linear peptide trace, cyan – monocyclic peptide trace, grey – bicyclic peptide trace, pink – tricyclic peptide trace.

We next wished to investigate the inclusion of OxyA into the OxyB/OxyE coupled turnover (Table 1), as this presents the first opportunity to investigate potential tricyclisation of a linear GPA precursor peptide *in vitro* (due to the lack of catalytic activity of the OxyC homologue from teicoplanin biosynthesis)^27^,^28^. Under conditions where OxyB, OxyE and OxyA were all included at the same time point and using the natural peptide diastereomer T7P(l-Hpg_7_) as a substrate we could identify tricyclic product, albeit at low levels of conversion (Fig. 7, left panel). A comparison of the LCMS traces generated by the coupled OxyB/OxyA^27^,^29^ and OxyB/OxyE reaction indicates that the major product identified corresponds to the bicyclic product resultant from OxyB and OxyA catalysis: this indicates that under these experimental conditions OxyE is unable to compete effectively with OxyA for monocyclic *peptidyl*-PCP-X substrate and furthermore that OxyE is unable to cyclise the bicyclic product produced by OxyB/OxyA catalysis. We then altered our experimental design to perform an initial pre-incubation of OxyB and OxyE with the *peptidyl*-PCP-X substrate, followed by later addition of OxyA. Under these conditions we could see a doubling of the amount of tricyclic peptide identified, with around 15% of the total peptide able to be cyclised by the actions of OxyB, OxyE and OxyA (Fig. 7, right panel).

## Discussion

In this work we have been able to demonstrate the first reports of OxyE activity from the peptide cyclisation cascade of GPA biosynthesis. Crucially, this activity has been shown to rely upon the presence of the X-domain in the substrate along with the *peptidyl*-PCP, whilst the interaction of the X-domain with OxyE could be demonstrated under gel filtration conditions using lower salt concentrations than was needed for the other Oxy enzymes^28^. This result harmonises the role of the X-domain as the general recruitment platform for the Oxy enzymes during GPA biosynthesis and supports the cyclisation of the precursor peptide *in vivo* as occurring on the final NRPS module, and hence as a heptapeptide. The activity of OxyE depends upon the initial introduction of the C-*O*-D ring in the peptide by OxyB, a result that matches what has been found from *in vivo* gene disruption experiments^13^. Our turnover results indicate that OxyE is unable to effectively install the F-*O*-G ring in the monocyclic peptide precursor when OxyA is also present, although a sequential introduction of OxyE followed by OxyA will allow production of a tricyclic peptide, presumably due to the order of activity OxyB − > OxyE − > OxyA; such a reaction order is in agreement with that identified from *in vivo* experiments^13^. Our experiments use a peptide substrate that contains an altered residue (Hpg rather than Dpg) at position 3 of the peptide and thus the F-*O*-G ring formed by OxyE is altered when compared to the natural ring – this could be a contributing factor to the lack of OxyE activity when OxyA is also present. The fact that OxyE activity is high when OxyA is not present indicates that the introduction of the unusual F-*O*-G ring only becomes challenging if the D-*O*-E ring is already present in the peptide, possibly due to the difficulty of orienting such a rigid bicyclic substrate correctly in the OxyE active site. The introduction of modified GPA crosslinks has been demonstrated to occur *in vivo*: OxyC can introduce the AB ring in a peptide where residue 7 had been switched from a Dpg to a Hpg residue, although in this case the larger ring size of the AB ring would be expected to reduce the strain of the bound peptide conformation and thus present less of a challenge for the Oxy enzyme involved^15^. Studies have suggested the presence of a natural Hpg_3_ residue in a type-IV GPA cluster, which would match the structure of the peptide we have used in this study: however, this system has yet to be characterised in detail to support the assignment of a Hpg-residue at position 3 together with the presence of an F-*O*-G ring in the GPA product^44^. Given that the interaction and competition studies we have performed show that OxyE is not able to interact as strongly as OxyA with the X-domain^27^,^28^, it appears reasonable to suggest that further aspects of selectivity are involved in the natural cyclisation cascade not present in our experimental setup, although our results support the proposed *in vivo* order of the Oxy cascade (OxyE before OxyA)^13^. Within GPA biosynthesis, the role of OxyE as an optional member of the GPA cyclisation cascade makes its investigation of interest in terms of how this process fits within the constraints imposed by the installation of the three essential crosslinks. Whilst the sequence – and also structural – similarity of OxyE with OxyA is clear^32^,^45^,^46^, they display very different activities against peptide substrates. In particular, the selectivity displayed by OxyA for the correct peptide diastereomer is not found for the OxyE catalysed installation of the F-*O*-G ring, possibly due to the fusion of the OxyA D-*O*-E ring to the OxyB C-*O*-D ring. The need to introduce the Oxy enzymes at different time points during the cyclisation reaction also has implications for the use of these enzymes as biocatalysts in simplified *in vitro* turnover systems and indicates that the ability to introduce and also remove Oxy enzymes from the turnover mixtures is likely needed to maximise *in vitro* yield of complex, cyclised GPA peptides. Such knowledge will no doubt prove crucial in the future if the use of such P450s as biocatalysts is to be realised.

## Methods

### Peptide synthesis

The synthesis of *peptidyl*-CoA substrates (T7P(l/d-Hpg_7_), T7P(l-Hpg_7_), T7P(d-Hpg_7_)) and their characterisation were performed as previously described^26^,^29^,^37^.

### Protein constructs – Cloning, expression and purification

The cloning, expression and purification of the used proteins has been previously described. Briefly, PCP and PCP-X (Tcp12, Uniprot ID Q70AZ6)^28^ were derived from codon optimized genes obtained from Eurofins Genomics MWG. After amplification the respective PCR fragments were cloned into a vector which enabled the expression of the proteins with IgG-binding B1 domain of *Streptococcus* (GB1) as an N-terminal fusion partner under the control of a T7-promotor^47^. The purification was performed via a two-step affinity chromatography protocol using the N-terminal hexahistidine-tag and a C-terminal Strep-tag followed by a final size exclusion chromatography as previously described. The Oxy proteins were amplified from genomic DNA (OxyB: Tcp20, Uniprot ID Q70AY8^21^; OxyA: Tcp18, Uniprot ID Q6ZZI8^29^; OxyE: Tcp19, Uniprot ID Q6ZZI7^32^) and cloned into a pET151D-TOPO vector (Life Technologies). The proteins were expressed with an N-terminal hexahistidine-tag and a V5 epitope followed by TEV protease cleavage site under the control of a T7-promotor. The purification procedure included metal affinity chromatography, TEV-cleavage of the N-terminal hexahistidine-tag, anion exchange chromatography and a final size exclusion chromatography step as previously described for OxyB^21^.

### Determining protein-protein interactions

The interaction between OxyE and the X-domain was analysed by analytical size exclusion chromatography; the proteins were mixed in a 1:3 ratio (33 μM OxyE and 100 μM PCP-X) in gel filtration running buffer (50 mM Hepes pH7.0, 50 mM NaCl) in a total volume of 120 μL. In addition, one control sample was prepared containing only OxyE. The samples were incubated for 30 min at RT. The interaction of the proteins was analysed on a 24 mL Superose 12 10/300 GL column (GE Healthcare) connected to an Äkta FPLC system. The samples were centrifuged (15 min, 4 °C, 12,000 × g) and 100 μL of the sample was loaded onto the column using a 0.1 mL injection loop. The flow rate applied was adjusted to 0.8 mL/min and the elution profile of the proteins was recorded at 280 and 415 nm.

### PCP-loading reaction

Loading of the PCP containing proteins (PCP or PCP-X) with *peptidyl*-CoA substrates (T7P(d/l-Hpg7)-CoA, T7P(d-Hpg7)-CoA, T7P(l-Hpg7)-CoA) was catalysed by an engineered phosphopantetheinyl transferase from *B. subtilis* (Sfp R4-4)^36^. PCP proteins (60 μM) were incubated with a 3-fold molar excess of *peptidyl*-CoA and 6 μM Sfp in PCP-loading buffer (50 mM Hepes pH 7.0, 50 mM NaCl, 10 mM MgCl_2_) for 1 h at 30 °C. Following the loading reaction, the excess of free *peptidyl*-CoA was removed from the loading reaction by a concentration dilution (4 × 1:5 dilution) procedure using 50 mM Hepes pH 7.0, 50 mM NaCl low salt buffer (0.5 mL Ultracentrifugal filters, 10,000 MWCO, Merck Millipore). The generated *peptidyl*-PCP constructs were used immediately after the PCP-loading reaction as substrates for the P450 activity assays.

### P450 activity assays

For the standard activity reaction 50 μM *peptidyl*-PCP substrate were mixed with one or more Oxys (2 μM) in 50 mM Hepes pH 7.0, 50 mM NaCl. The electrons required for the oxygenation reaction were obtained from NADH (2 mM). In addition 5 μM palustrisredoxin B (PuxB variant A105V) and 1 μM palustrisredoxin reductase (PuR) from *Rhodopseudomonas palustris*^41^ were added to the reaction in order to mediate electron transfer from NADH to the Oxys. The NADH was regenerated throughout the reaction through the addition of β-D-glucose (0.33% (w/v)) and glucose dehydrogenase (9 U/mL). The reactions were started through the addition of NADH and incubated for 1 h at 30 °C under gentle shaking. For the coupled OxyB/OxyA/OxyE assay two different sets of reactions were performed. In the first set of reactions all P450s were added at the same time to the reaction. In a second set of reactions OxyB and OxyE were pre-reacted for 30 min and 30 °C with the substrate before OxyA was added to the reaction. The reactions were halted and the peptides were cleaved from the carrier protein upon addition of methylhydrazine (for T7P(d/l-Hpg7)-PCP/-PCP-X substrates, 23,000-fold molar excess over substrate) or methylamine (for all other substrates, 32,000-fold molar excess over substrate). After incubation for 15 minutes, the solution was neutralised through the addition of formic acid (diluted in water) and the peptides were purified by solid phase extraction using Strata-X-33 polymeric reversed phase columns (30 mg/mL, Phenomenex). The crosslinking state of the peptide was analysed via HPLC-MS by recording the masses for the different peptide species using single ion monitoring (SIM) in negative mode. The HPLC separation was performed using a Waters XBridge BEH 300 Prep C18 column (particle size: 5 μm, 4.6 × 250 mm) with the following conditions: 0–4 min 95% water + 0.1% formic acid (solvent A), 4–4.5 min up to 15% HPLC-grade acetonitrile + 0.1% formic acid (solvent B), 4.5–25 min up to 50% solvent B; flow rate 1 mL/min. After integration of the signals obtained for the different peptide species the P450 activities were calculated based on the percentage of cyclised peptide relative to the respective substrate. In the reactions containing all three P450s the identity of the crosslinks cannot be explicitly defined by the retention time of the peptide compound. Hence, the data are expressed as the amount of the respective crosslinking species relative to the total amount of peptide detected.

### P450 competition assay

To analyse potential competition of OxyB and OxyE for the *peptidyl*-PCP-X substrate the activity of OxyB towards T7P(l-Hpg7)-PCP-X was determined in the presence of OxyE. Thus, OxyB (3 μM) and OxyE (3 μM) were either added at the same time to the substrate (2.5 μM) (reaction 2) or one of the Oxys was pre-incubated with the substrate (30 min at RT, reaction 1 and 3) before the reaction was initiated. The crosslinking reaction was performed in 50 mM Hepes pH 7.0, 50 mM NaCl low salt buffer, additionally containing PuxB (15 μM) and PuR (3 μM). Before the reaction the redox partners (PuxB and PuR) and the NADH regeneration system (0.33% (w/v) β-D-glucose, 9 U/mL glucose dehydrogenase) were pre-incubated in reaction buffer at 30 °C. In order to start the reaction, *peptidyl*-PCP-X, P450s and NADH were added at specific time points to the reaction mixture. First, the *peptidyl*-PCP-X (either alone or in a mixture with one of the Oxys) was added to the reaction and incubated for 30 seconds. Next the second P450 (reaction 1: OxyE, reaction 3: OxyB) or a mixture of both P450s (reaction 2) was added and after 10 seconds the reaction was started through the addition of 2 mM NADH. The reactions were quenched and the peptides were cleaved from the PCP-X protein after 5 seconds by pipetting 105 μL of the reaction mixture into 15 μL of methylamine. The peptide work-up and analysis was performed as described for the standard turnover reaction.

## Additional Information

**How to cite this article**: Peschke, M. *et al*. F-*O*-G Ring Formation in Glycopeptide Antibiotic Biosynthesis is Catalysed by OxyE. *Sci. Rep.* **6**, 35584; doi: 10.1038/srep35584 (2016).

## Supplementary Material

Supplementary Information

## Figures and Tables

**Figure 1:**
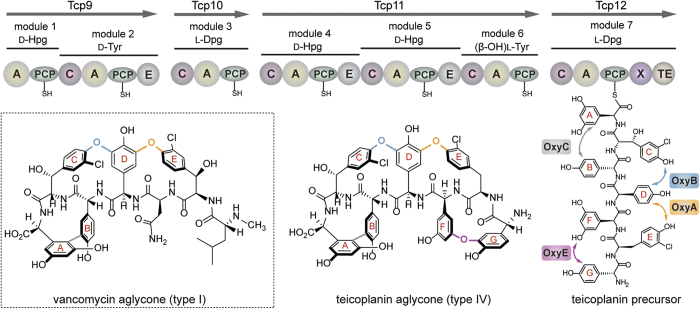
Schematic representation of glycopeptide antibiotic biosynthesis by a combination of non-ribosomal peptide synthesis and Cytochrome P450-mediated (Oxy enzyme mediated) side chain crosslinking; examples of a type I (vancomycin) and type IV (teicoplanin) glycopeptide antibiotic aglycone are shown. Non-ribosomal peptide synthetase domains are indicated using the following nomenclature. A (adenylation), PCP (peptidyl carrier protein), C (condensation), E (epimerisation), X (P450 recruitment) and TE (thioesterase). Incorporated amino acids are indicated above the modules: Hpg (4-hydroxyphenylglycine), Dpg (3,5-dihydroxyphenylglycine). Oxy enzyme crosslinking is shown for the teicoplanin precursor peptide.

**Table 1:** Results of coupled OxyB/OxyA/OxyE turnovers.

Oxy enzymes	peptidyl-PCP-X	monocyclic peptide [%]^a^	bicyclic peptide [%]^a^	tricyclic peptide [%]^a^
B/A/E	T7P(L-Hpg_7_)	17 ± 1	37 ± 2	8 ± 1
B/A/E	T7P(D-Hpg_7_)	26 ± 2	39 ± 1	4 ± 1
B/E/A^b^	T7P(L-Hpg_7_)	17 ± 2	33 ± 2	15 ± 1
B/E/A^b^	T7P(D-Hpg_7_)	26 ± 3	34 ± 3	7 ± 1

^a^Data are expressed as the percentage of the specific peptide relative to the total amount of detected peptide. Monocyclic peptide generated by the activity of OxyB, bicyclic peptide generated by the additional activity of OxyA or OxyE, tricyclic peptide generated by the combined activity of OxyB, OxyA and OxyE. Results obtained from triplicate experiments, ± standard deviation.

^b^OxyA added to the reaction after an initial 30-minute incubation of OxyB_tei_ and OxyE_tei_ with the substrate.
