# Supplementary material for: F-O-G Ring Formation in Glycopeptide Antibiotic Biosynthesis is Catalysed by OxyE
Source: Sci Rep. 2016 Oct 18;6:35584. doi: 10.1038/srep35584 (PMC5067714; doi:10.1038/srep35584)
Supplement: Supplementary Information [file srep35584-s1.pdf]

F-*O*-G Ring Formation in Glycopeptide Antibiotic Biosynthesis is Catalysed by OxyE

Madeleine Peschke, [a] Clara Brieke [a] and Max J. Cryle* [a,b,c]

a. Department of Biomolecular Mechanisms, Max Planck Institute for Medical Research, Jahnstrasse 29, 69120 Heidelberg, Germany.

b. EMBL Australia, Monash University, Clayton, Victoria 3800, Australia.

c. The Monash Biomedicine Discovery Institute, Department of Biochemistry and Molecular Biology and ARC Centre of Excellence in Advanced Molecular Imaging, Monash University, Clayton, Victoria 3800, Australia.

* Correspondence should be addressed to: max.cryle@monash.edu

Supplementary Information

SI Table 1. Results of OxyE and coupled OxyB/OxyE turnover reactions

Oxy enzymes	peptide	carrier	OxyB activity [%][a]	OxyE activity [%][a]
OxyE	T7P(D/L-Hpg_7)	PCP	-	4 ± 1
OxyB/OxyE	T7P(D/L-Hpg_7)	PCP	31 ± 1	1 ± 1
OxyE	T7P(D/L-Hpg_7)	PCP-X	-	5 ± 1
OxyB/ OxyE	T7P(D/L-Hpg_7)	PCP-X	71 ± 2	42 ± 1
OxyB/ OxyE	T7P(L-Hpg_7)	PCP-X	70 ± 3	72 ± 1
OxyB/ OxyE	T7P(D-Hpg_7)	PCP-X	82 ± 1	57 ± 1

[a] The Oxy activities expressed as the percentage of cyclized peptide relative to the respective substrate. Results are obtained from triplicate experiments, ± standard deviation.

SI Table 2. Results of OxyB/OxyE competition experiments

Reaction[a]	OxyB activity [%][b]
1 (OxyB only)	56 ± 7 [1]
2 (OxyB only)	51 ± 4 [1]
1	37 ± 5
2	28 ± 4
3	22 ± 5

[a] Assignment of the reactions according to Figure 6; reaction 1 addition of OxyB before OxyE, reaction 2 simultaneous addition of OxyB and OxyE and reaction 3 addition of OxyE before OxyB.

[b] Data are expressed as the percentage of the monocyclic peptide relative to the total amount of detected peptide. Results obtained from triplicate experiments, ± standard deviation.

References

1 Peschke, M., Haslinger, K., Brieke, C., Reinstein, J. & Cryle, M. Regulation of the P450 oxygenation cascade involved in glycopeptide antibiotic biosynthesis. *J. Am. Chem. Soc.* **138**, 6746-6753 (2016).
